# Prior exposure to immunogenic peptides found in human influenza A viruses may influence the age distribution of cases with avian influenza H5N1 and H7N9 virus infections

**DOI:** 10.1017/S095026881900102X

**Published:** 2019-06-13

**Authors:** N. Komadina, S. G. Sullivan, K. Kedzierska, S. M. Quiñones-Parra, K. Leder, J. McVernon

**Affiliations:** 1 WHO Collaborating Centre for Reference and Research on Influenza, Royal Melbourne Hospital at the Peter Doherty Institute for Infection and Immunity, Victoria, 3000, Australia; and School of Public Health and Preventive Medicine, Monash University, 553 St Kilda Road, Melbourne, Australia; 2 WHO Collaborating Centre for Reference and Research on Influenza, Royal Melbourne Hospital and the Doherty Department, University of Melbourne at the Peter Doherty Institute for Infection and Immunity, Victoria, 3000, Australia; and Melbourne School of Population and Global Health, The University of Melbourne, Melbourne, 3000, Australia; 3 Department of Microbiology & Immunology, University of Melbourne at the Peter Doherty Institute for Infection & Immunity, Victoria, 3000, Australia; 4 Department of Molecular Biology, University of California, San Diego, California, USA; 5 School of Public Health and Preventive Medicine, Monash University, 553 St Kilda Road, Melbourne, Australia; and Victorian Infectious Disease Service, Royal Melbourne Hospital at the Peter Doherty Institute for Infection and Immunity, Victoria, 3000, Australia; 6 Victorian Infectious Diseases Reference Laboratory Epidemiology Unit, Royal Melbourne Hospital and the Doherty Department, University of Melbourne at the Peter Doherty Institute for Infection and Immunity, Victoria, 3000, Australia; Melbourne School of Population and Global Health, The University of Melbourne, Melbourne, 3000, Australia and the Murdoch Children's Research Institute, Melbourne, 3000, Australia

**Keywords:** Avian flu, immunogenic peptides, influenza A, H5N1, H7N9

## Abstract

The epidemiology of H5N1 and H7N9 avian viruses of humans infected in China differs despite both viruses being avian reassortants that have inherited six internal genes from a common ancestor, H9N2. The median age of infected populations is substantially younger for H5N1 virus (26 years) compared with H7N9 virus (63 years). Population susceptibility to infection with seasonal influenza is understood to be influenced by cross-reactive CD8+ T cells directed towards immunogenic peptides derived from internal viral proteins which may provide some level of protection against further influenza infection. Prior exposure to seasonal influenza peptides may influence the age-related infection patterns observed for H5N1 and H7N9 viruses. A comparison of relatedness of immunogenic peptides between historical human strains and the two avian emerged viruses was undertaken for a possible explanation in the differences in age incidence observed. There appeared to be some relationship between past exposure to related peptides and the lower number of H5N1 virus cases in older populations, however the relationship between prior exposure and older populations among H7N9 virus patients was less clear.

## Introduction

The influenza viruses cause significant respiratory tract infections and economic burden globally, due to yearly epidemics and intermittent pandemics [1, 2]. Influenza A pandemics have arisen as a result of zoonotic viruses gaining the ability to transmit in humans usually by reassortment. Wild bird aquatic species such as ducks, geese and swans are the prime reservoirs for influenza A viruses (IAVs), shedding the virus through their droppings. Domestic poultry can become infected either directly or via incidental contact with contaminated feed or water and if introduced into poultry the virus spreads rapidly throughout flocks [3]. Transmission of influenza viruses from wild aquatic birds to humans is relatively rare due to low exposure. However, once the virus has infected domestic poultry, the risks of human exposure and transmission increase [4].

The first case of direct transmission of the avian H5N1 virus from domestic poultry to humans was documented in Hong Kong in 1997. The virus later re-emerged in humans in mainland China in 2003 [5, 6]. Avian H7N9 influenza virus was first detected in humans from Anhui province, China in 2013 [7]. The emergence of the H5N1 viruses in humans was generally preceded by highly pathogenic outbreaks in farmed poultry, whereas for the H7N9 virus it appears that there was a silent introduction of the wild bird avian influenza into domestic poultry before the virus was transmitted to humans [8, 9]. The 1997 and 2003 H5N1 as well as the 2013 H7N9 influenza viruses were avian reassortants that had inherited all six internal genes from an avian H9N2 virus common ancestor [10]. Although both H5N1 and H7N9 viruses cause severe disease in humans, the epidemiology in humans differs for each virus [9].

## Epidemiology of H5N1 virus in different populations

Since re-emergence of the H5N1 virus in China in 2003, genetic variants have been isolated from humans throughout South East Asia, the Middle East, Africa and Europe [8]. Comparisons of the epidemiological characteristics of different populations however, have shown some differences amongst countries [9]. The case fatality risk of those admitted to hospitals varied amongst countries with considerably lower fatality risk in Vietnam (39%), and higher risk in China (70%) [9]. A comparison of H5N1 virus patients in China and Vietnam identified differences in clinical symptoms on admission, despite similarities in demographic characteristics, underlying medical conditions and behavioural risks suggesting viral factors may impact severity of disease [11]. Differences observed in H5N1 virus case fatality risks may further have been influenced by health sector variables including access (time to admission) and the level of available in-hospital care [12, 13]. Inconsistent gender biases in those infected have also been observed amongst countries [14] and in some settings differences in preponderance of male or female cases have been attributed to local cultural habits [15].

## Avian influenza H5N1 and H7N9 virus infections in humans in China

Given the variety of factors that may affect observed epidemiology between countries, we focus here on a comparison of human cases of avian H5N1 and H7N9 influenza viruses restricted to those occurring in China. A total of 53 laboratory confirmed human cases of H5N1 avian influenza virus were reported from 2003 to 2016 and 779 confirmed human H7N9 virus cases from 2013 to 2016. The H5N1 virus cases were found throughout mainland China whereas H7N9 virus was initially more concentrated in the Yangtze river delta [9]. The incidence of H7N9 virus in the year of emergence, 2013, was almost 10-fold greater than that of H5N1 virus in 2003, the year of its emergence [9]. The viruses also had differing age specific epidemiology with individuals with H5N1 virus infection presenting at a median age of 26 years and H7N9 virus, 63 years (Table 1) [11, 16]. For H7N9 virus there was a clear predominance of males in China but there was no evidence of gender differences in human H5N1 virus cases [9, 14]. As Table 1 further shows case fatality rates for H5N1 virus were also substantially higher than for H7N9 virus cases [14, 17]. There are some similarities in probable routes of infection with the majority of cases reporting some contact with poultry prior to onset of illness [16, 18]. Similar types of influenza-like illness are associated with both subtypes as is the suspicion of non-sustained human-to-human transmission having occurred in a limited number of instances [8, 18].

**Table 1.** Comparative epidemiology of laboratory confirmed cases [9, 43]

Epidemiology	H5N1	H7N9
Median age, years	26	63
Number of cases in first year of outbreak	50	373
Gender differences	None	Male: 67%
Case fatality rate	60%	22%
Presence of underlying medical conditions	12%	45%
Urban residents	44%	72%
Exposure to poultry	71%	77%

## Cross-protection due to conserved CD8+ T cell epitopes

Cowling *et al*. speculated that the different age distribution of H5N1 and H7N9 virus cases was associated with immunity from different seasonal influenza exposure histories [9]. Studies have provided evidence that haemagglutinin (HA) stalk-specific immunity is gained from an individual's first contact with IAVs providing lifelong protection against severe illness by novel, yet related HA subtypes and this HA imprinting is thought to be the driver behind the age-specific differences noted between H5N1 and H7N9 infections [25]. Gostic *et al*. have further suggested that CD8+ T cell responses may also play a role in age-specific differences.

Following influenza infection, CD8+ T cells responses directed towards immunogenic peptides derived from the NP, M1 and PB1 proteins can provide a level of cross-protection against influenza disease [22, 23], with implications for population immunity against seasonal influenza [19]. Pre-existing CD8+ T cell protection has been reported against the pandemic H1N1pdm09 influenza strain [20]. Experimental studies have identified a number of conserved CD8+ T cell immunogenic peptides between seasonal, pandemic and avian influenza viruses including H7N9 [21]. It has been hypothesised that previous exposure to these peptides may have an influence on age-related mortality patterns seen for H5N1 and H7N9 viruses [24]. Thus, despite the absence of prior circulation of H5N1, H7N9 or H9N2 viruses in the human population, conserved CD8+ T-cell peptides may afford influenza cross-strain protection.

This study compares CD8+ T cell immunogenic peptide sequences in the NP, PB1 and M1 proteins of the Chinese H5N1 and H7N9 avian influenza viruses with matched sequences from H1N1, H2N2, H3N2 and pandemic H1N1 influenza viruses that circulated from 1918 to 2013. We hypothesise that differences in relatedness of peptides between the historical human strains and the two avian-emerged strains could in part explain the differences in age–incidence of disease observed due to H5N1 and H7N9 viruses.

## Methods

### Selection of reference peptides

Sequences of H5N1 and H7N9 influenza viruses that infected humans in China were sourced from the Global Initiative on Sharing all Influenza Data (GISAID EpiFlu™ database, www.gisaid.org) [26].

Consensus sequence was determined for each protein of interest for both subtypes using Geneious V10.0.9 (www.geneious.com). A total of 73 NP, 33 PB1 and 37 M1 peptides were selected from the IEDB (www.iedb.org). Our search filtered only for experimentally defined epitopes that were identified using T-cell assays. Diversity amongst the peptides found in the NP, PB1 and M1 proteins of the H5N1 and H7N9 viruses was calculated as the proportion of viruses per peptide that matched the reference peptide consensus. These were then plotted using R v3.4 (www.cran-r-project.org) [27].

### Data preparation

Data were prepared using the alignment tool in Geneious V10.0.9 (www.geneious.com). Protein sequences were excluded if they were: derived from laboratory adapted or generated viruses; duplicates; did not span the full range of peptides and were incomplete. Sequences derived from clinical specimens, lowest passage history or passaging in cell rather than eggs was selected preferentially from available duplicate sequences to minimise the occurrence of mutations due to adaptation in the regions of interest. Data were trimmed to begin with the first methionine of the NP, M1 and PB1 genes. A unique sequential number was inserted into the fasta header to maintain a consistent, sequential order when tabulating the peptides of the test data.

### Analysis of historical peptide sequences

These reference peptide sequences were compared with the corresponding peptide sequences of historical subtypes; i.e. H1N1 (pre-1957 and post-1977), H2N2, H3N2 and H1N1pdm09 viruses. The proportion of sequences identical to the reference peptide for each peptide, each subtype and each year was calculated. For example, reference peptide NP_17–25_ is compared with peptide NP_17–25_ in each of the subtypes (Figs S2–S4). The proportion of identical sequences was generally aggregated into 5 year blocks from 1933 to 2013. Prior to 1933, only one historical H1N1 sequence was available for comparison. Analyses were performed using Stata V12.1 (www.stata.com) [28]. Peptide diversity per protein per peptide per year per subtype was calculated and plotted using R. v3.4, Figure S1 (www.cran.r-project.org) [27].

### Patient data

**Fig. 1.**
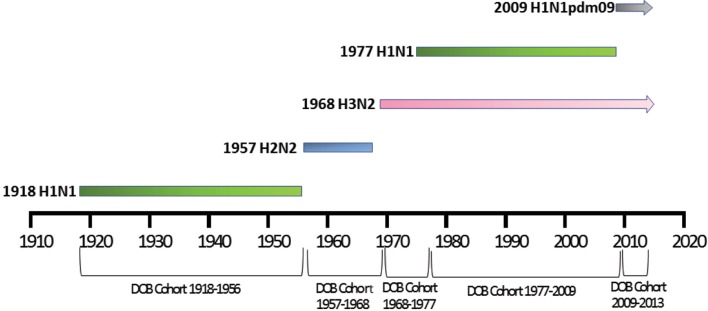
Selected date of birth cohorts defined in relation to the emergence of novel influenza viruses in the human population over the time-period of interest, 1918–2013.

Patient meta-data were available for some of the human H5N1 and H7N9 viral sequences on the GISIAD database [26]. Where age at infection was available in the patient meta-data, mean and median ages of infection with either virus were calculated. In addition the year of birth was estimated, allowing reporting of the proportion of patients infected with each virus who were born within specific birth cohorts. These cohorts were defined according to the emergence of novel influenza viruses to which individuals may have been exposed: 1918–1956 (H1N1), 1957–1968 (H2N2), 1968–1977 (H3N2), 1977–2009 (re-emergence of H1N1, co-circulation with H3N2) and 2009–2013 (emergence of H1N1pdm09, co-circulation with H3N2), Figure 1.

## Results

### Sequence data

A total of 2792 H1N1, 224 H2N2, 14 972 H3N2 and 10 472 H1N1pdm09 sequences were identified in GISAID. The number of available sequences for each of the NP, PB1 and M1 immunogenic peptides per subtype is shown in the Supplementary data, Table S1.

### H5N1 and H7N9 reference data

For the H7N9 viruses the following protein sequences were identified: 342 NP, 348 PB1 and 353 MP. Available H5N1 viral sequences identified were fewer, resulting in the identification of 41 NP, 40 PB1 and 42 MP sequences. Peptide diversity within the H5N1 and H7N9 peptides was calculated and a comparison of means show that for the H5N1 peptides the mean for the three genes ranged from 96% to 98% and for the H7N9 96% to 99%. The results of the minimum, maximum and the mean per gene are given in Table 2.

**Table 2.** Proportion of peptides identical to the corresponding reference peptides

Subtype	Gene	Minimum (%)	Maximum (%)	Mean (%)
H5N1	NP	50	100	96
	PB1	87	100	98
	M1	81	100	98
H7N9	NP	77	100	96
	PB1	76	100	98
	M1	96	100	99

### Patient data

**Fig. 2.**
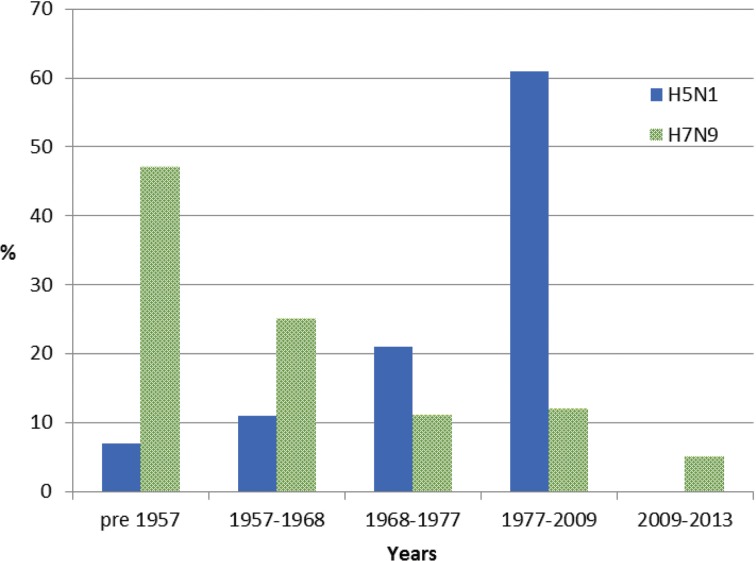
Year of birth of individuals from whom H5N1 and H7N9 viruses were isolated between 2003 and 2016. Birth years are grouped into cohorts, separated by the year of emergence of pandemic influenza strains. Individuals experiencing H7N9 infection (green) were substantially older than those infected with H5N1, the majority of whom were born in the period 1977–2009.

For meta-data associated with the 46 H5N1 viral sequences sourced from GISAID [26] used in this study, patient age was recorded for 57%. The mean and median ages at infection were 29 and 28 years, respectively. Of these individuals, 7% were born before 1957, 11% between 1957 and 1968, 21% between 1968 and 1977 and 61% between 1977 and 2009, with none after 2009 (Fig. 2). Gender was provided for 53% of the sequenced isolates and 58% were reported as male. Patient outcome was also reported for 27% of the isolate sequences, with 69% of these reported as deceased.

For meta-data available with all 478 H7N9 viral sequences sourced from GISAID [26], age was available for 90% of sequenced isolates with a mean age of 53 years and a median age of 56 years. Of these individuals, 47% were born before 1957, 25% between 1957 and 1968, 11% between 1968 and 1977, 12% between 1977 and 2009 and 5% after 2009 (Fig. 2). Gender was provided for 98% of sequences and 67% were male. Patient outcome was provided for 12% of cases, of whom 3% were reported as deceased.

### Comparison of sequence differences in H5N1 and H7N9 viral NP, PB1 and M1 immunogenic peptides

#### NP immunogenic peptides

Of the 73 NP peptides, only 12 were found to differ by one or two amino acids between H5N1 and H7N9 viruses (Table 3). These 12 peptides did not include any of the peptides previously described as highly conserved in human influenza viruses [21, 29]. Two of the 12 peptides have been associated with prominent CD8+ T-cell responses in humans, NP_44–52_ restricted by HLA-A*0101 and NP_404–413_ restricted by HLA-B*1501 [30, 31]. These peptides also differed between the H5N1 and H7N9 viruses in either the amino acids in the anchor residue, T52N change in NP_44–52_ or in the position adjacent to the anchor residue, I406V change in NP_404–413_. However, it is not known whether these single amino acid differences, or the novelty of peptides, are associated with any functional significance that might influence cross-protection.

**Table 3.** Sequence variation of one or more amino acids between the H5N1 and H7N9 immunogenic peptides of interest in the NP

Position	H5N1 (*n* = −41)	H7N9 (*n* = 342)
**37–54**	GRFYIQMCTELKLSD**Y**EG	GRFYIQMCTELKLSD**N**EG
**40–57**	YIQMCTELKLSD**Y**EGRLI	YIQMCTELKLSD**N**EGRLI
**44–52**^a^	CTELKLSD**Y**	CTELKLSD**N**
**48–56**	KLSD**Y**EGRL	KLSD**N**EGRL
**169–186**	GSTLPRRSGAAGAAVKG**V**	GSTLPRRSGAAGAAVKG**I**
**170–186**	STLPRRSGAAGAAVKG**V**	STLPRRSGAAGAAVKG**I**
**337–353**	AFEDLRVSSFIRGTR**V**V	AFEDLRVSSFIRGTR**M**V
**373–381**	**T**M**D**SNTLEL	**A**M**E**SNTLEL
**373–390**	**T**M**D**SNTLELRSRYWAIRT	**A**M**E**SNTLELRSRYWAIRT
**397–414**	NQQRASAGQ**I**SVQPTFSV	NQQRASAGQ**V**SVQPTFSV
**404–413**^a^	GQ**I**SVQPTFS	GQ**V**SVQPTFS
**404–414**	GQ**I**SVQPTFSV	GQ**V**SVQPTFSV

Differences are shown as bold with known anchor points underlined [30].

a Peptides previously identified as prominent.

#### PB1 and M1 immunogenic peptides

Of the 33 PB1 peptides, six peptides were found to differ in sequence between the H5N1 and H7N9 viruses (Table 4). Of the 37 M1 peptides, 10 different sequences were identified between the H5N1 and H7N9 viruses (Table 5).

**Table 4.** Sequence variation of one or more amino acids between H5N1 and H7N9 immunogenic peptides of interest in the PB1 protein

Position	H5N1 (*n* = 40)	H7N9 (*n* = 348)
**41–49**	DTVNRTH**Q**Y	DTVNRTH**K**Y
**166–174**	FLKDVM**E**SM	FLKDVM**D**SM
**254–262**	FVE**T**LARSI	FVE**A**LARSI
**257–265**	**T**LARSICEK	**A**LARSICEK
**406–422**	GMMMGMFNMLSTVLGV**S**	GMMMGMFNMLSTVLGVS**I**
**566–574**	TQIQTRR**S**F	TQIQTRR**A**F

Differences in amino acids are shown as bold.

**Table 5.** Sequence variation of one or more amino acids between the H5N1 and H7N9 immunogenic peptides of interest in M1 protein

Position	H5N1 (*n* = 42)	H7N9 (*n* = 353)
**17–31**	SGPLKAEIAQ**K**LEDV	SGPLKAEIAQ**R**LEDV
**27–35**	**K**LEDVFAGK	**R**LEDVFAGK
**29–37**	EDVFAGKN**T**	EDVFAGKN**A**
**33–49**	AGKN**T**DLEALMEW**L**KTR	AGKN**A**DLEALMEW**I**KTR
**34–53**	GKN**T**DLEALMEW**L**KTRPILS	GKN**A**DLEALMEW**I**KTRPILS
**40–57**	EALMEW**L**KTRPILSPLTK	EALMEW**I**KTRPILSPLTK
**95–112**	**R**AVKLYKKLKRE**I**TFHGA	**K**AVKLYKKLKRE**M**TFHGA
**99–109**	LYKKLKRE**I**TF	LYKKLKRE**M**TF
**239–248**	AYQ**K**RMGVQ**M**	AYQ**N**RMGVQ**L**
**241–252**	Q**K**RMGVQ**M**QRFK	Q**N**RMGVQ**L**QRFK

Differences in amino acids are shown as bold.

### Comparison of H5N1 and H7N9 reference peptides with historical peptides circulating between 1918 and 2013 in human influenza viruses

#### Peptide sequence diversity analysis

The diversity amongst all the immunogenic peptides found in the NP, PB1 and M1 proteins of the H1N1, H2N2, H3N2 and H1N1pdm09 isolates used in the study was calculated and plotted per year, Supplementary Material, Figure S1. The level of divergence amongst the peptides was similar across all subtypes. In general each peptide had one dominant variant with the majority of the remaining variants being single variants. The greater the number of sequences available the greater the within-peptide diversity was noted. This was most noticeable with the outbreak of the H1N1 pandemic virus in 2009 which had the highest number of sequences available for a single year of any of the subtypes and also exhibited the greatest diversity within the peptides, Table S1 and Figure S1.

#### NP immunogenic peptides

**Fig. 3.**
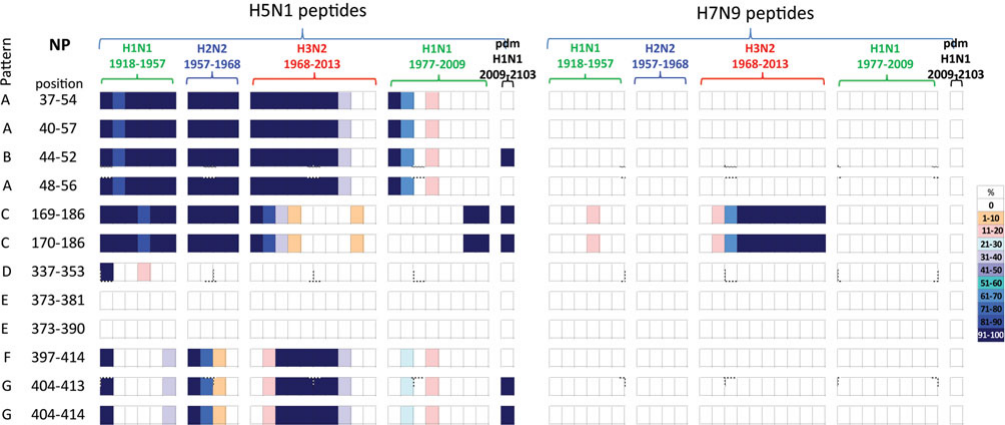
Comparisons of the temporal circulation patterns of 12 NP peptides with differential sequences between the H5N1 and H7N9 virus immunogenic peptides. Data for the H5N1 peptides are shown on the left, whilst H7N9 peptides are shown on the right. Variation of one or more amino acids between H5N1 and H7N9 peptides of interest is shown in Table 3. The plots for each of the immunogenic peptides of interest represent the percentage of the corresponding sequences found in the human viruses that match the H5N1 or H7N9 consensus sequence. The H7N9 virus peptides display a greater degree of novelty (83%) with only two peptides having circulated in H3N2 viruses after emerging in 1977. For the H5N1 virus peptides, only three (25%) were novel to the human population with the remainder circulating at various levels in H1N1, H2N2 and H3N2 viruses over the study period. Circulation of the H5N1 virus peptides differed in the H1N1pdm viruses as there was greater novelty with only 42% found circulating in these viruses. For simplicity, the H5N1 virus peptides with similar temporal patterns have been grouped together with each pattern type assigned a letter (A–G).

A comparison of the 12 NP peptides with sequences that differed between H5N1 and H7N9 viruses revealed that two peptides in NP_373–390_, Table 3, were unique to both viruses, not having been seen in any human influenza subtypes over the preceding century, Figure 3.

Of the remaining 10 H7N9 viral peptides, only NP_169–186_ and NP_170–186_ peptides had previously circulated in humans, in H3N2 viruses isolated from the late 1970s onwards. The other eight peptides have unique sequences, not previously circulating in human IAVs.

In contrast, the majority of the remaining 10 H5N1 viral peptide sequences examined had close homology with peptides identified from human viruses circulating over the study period. Of these 10 peptides, four peptides (NP_37–54/40–57/44–52/48–56_) were present in early H1N1, H2N2 and H3N2 strains from 1918 until 2000 when they were no longer found, Figure 3 (patterns A and B). These peptides were also briefly noted in the post-1977 H1N1 virus. One peptide re-emerged in the H1N1pdm09 viruses in 2009, Figure 3 (pattern B). Two peptides (NP_169–186/170–186_) were found continuously from 1918 to 1977 in early H1N2, H2N2 and H3N2 viruses but were present only in post-1977 H1N1 strains from late 2000s just prior to the replacement of the seasonal H1N1 viruses with the H1N1pdm09 viruses. These peptides were present in the H1N1pdm09 viruses, Figure 3 (pattern C). One peptide (NP_337–353_) was present on emergence of the H1N1 viruses in 1918, reappeared briefly in the 1940s then was no longer found in human influenza viruses, Figure 3 (pattern D). Two peptides (NP_373–381/373–390_) were novel, Figure 3 (pattern E), in the human population. The three remaining peptides (NP_397–414/404–413/404–414_) were present in newly emerged H1N1 (1918) and H2N2 (1957) strains but circulated only briefly before being replaced. They re-emerged in the 1968 H3N2 virus and on this occasion persisted over three decades. They have only been infrequently observed in post-1977 H1N1 viruses, Figure 3 (patterns F and G). Two of the three peptides re-emerged in the H1N1pdm09 viruses in 2009, Figure 3 (pattern G).

All NP peptide temporal circulation patterns are shown in Supplementary Material, Figure S2. Briefly for peptides that had the same sequences for both the H5N1 and H7N9 viruses, 12% were novel and not noted in human influenza viruses. Of the remaining peptides which had previously circulated in human influenza viruses, 8% were found in all human viruses, 31% and 30% were not found in H2N2 and H3N2 human viruses respectively with the remaining peptides found in varying levels and time points in the human IAVs.

#### PB1 immunogenic peptides

**Fig. 4.**
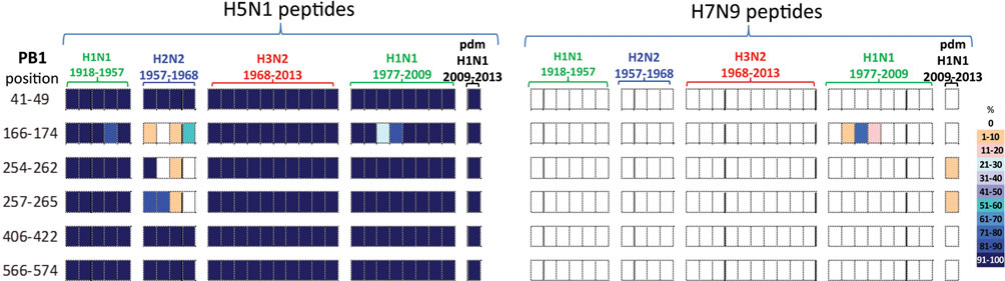
Comparisons of the temporal circulation patterns of six PB1 peptides with differential sequences between the H5N1 and H7N9 virus immunogenic peptides. Data for the H5N1 virus peptides are shown on the left, whilst H7N9 virus peptides are shown on the right. Variation of one or more amino acids between H5N1 and H7N9 peptides of interest is shown in Table 4. The plots for each of the immunogenic peptides of interest represent the percentage of the corresponding sequences found in the human viruses that match the H5N1 or H7N9 consensus sequence. All six of the H5N1 virus peptides had previously circulated in humans at high levels in all four influenza A subtypes. However, the H7N9 virus peptides were almost entirely novel in the human population with one peptide circulating in very low numbers between 1988 and 1991 in the post-1977 H1N1 viruses and further two peptides noted in very low numbers, ⩽10% circulating in the H1N1pdm viruses.

All six PB1 peptides whose sequence differed between H5N1 and H7N9 viruses, Table 4, also differed in their temporal circulation in the human H1N1, H2N2, H3N2 and H1N1pdm09 human viruses, Figure 4. Of the six H7N9 viral peptides, only one circulated in the human population prior to 2009 with (PB1_166–174_) appearing briefly in post-1977 seasonal H1N1 viruses. Two different peptides (PB1_254–262/257–265_) emerged with the H1N1pdm09 strain and were observed in <10% of H1N1pdm09 viruses circulating in humans from 2009 to 2013, Figure 4.

In contrast, the six H5N1 viral peptide sequences had a high degree of homology with the peptides identified in virus sequences available over the study period (Fig. 4). All six H5N1 viral peptides had >95% sequence homology with H1N1, H3N2 and H1N1pdm09 viruses. Three of the six peptides (PB1_41–49/406–422/566–574_) also had >95% sequence homology with the H2N2 viruses. Of the remaining three peptides, one (PB1_166–174_) was found sporadically in H2N2 viruses. Two peptides (PB1_254–262/257–265_) were found in the emergent H2N2 viruses in 1957, but by the mid-1960s had been replaced by a new variant.

All PB1 peptide temporal circulation patterns are shown in Supplementary Material, Figure S3. Briefly for peptides that had the same sequences for both the H5N1 and H7N9 viruses all peptides were found in varying levels and time points in circulating human influenza viruses, 70% of peptides were also found in almost all human influenza viruses with 4% not found in H1N1, H2N2 and pdmH1N1 viruses and a further 11% not found in pdmH1N1 human viruses.

#### M1 immunogenic peptides

**Fig. 5.**
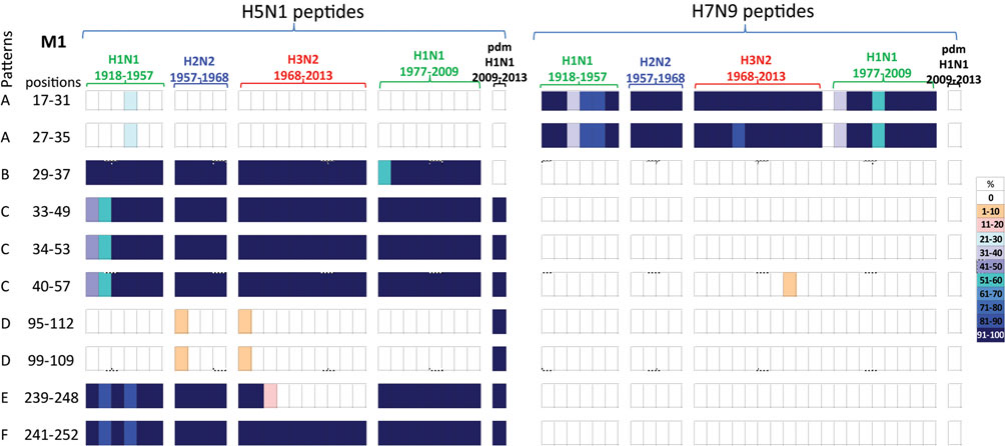
Comparisons of the temporal circulation patterns of 10 M1 peptides with differential sequences between the H5N1 and H7N9 virus immunogenic peptides. Data for the H5N1 virus peptides are shown on the left, whilst H7N9 virus peptides are shown on the right. Variation of one or more amino acids between H5N1 and H7N9 peptides of interest is shown in Table 5. The plots for each of the immunogenic peptides of interest represent the percentage of the corresponding sequences found in the human viruses that match the H5N1 or H7N9 consensus sequence. For the H5N1 virus peptides, 40% were novel with the remainder having previously circulated in the human population in a high level of viruses of all subtypes. For the H7N9 virus peptides, 80% were novel with only 20% having previously circulated in viruses in the human population. These H7N9 virus peptides were entirely novel in the H1N1pdm09 viruses. For simplicity, the H5N1 virus peptides with similar temporal patterns have been grouped together with each pattern type assigned a letter (A–F).

A comparison of the 10 M1 peptides with sequences that differed between H5N1 and H7N9 viruses, Table 5, revealed that the H7N9 viral peptides displayed greater novelty than the H5N1 viral peptides, Figure 5. Of the 10 H7N9 virus M1 peptides, two peptides (M1_17–31/27–35_) were noted circulating in the early H1N1, H2N2, H3N2 and post-1977 H1N1 viruses but not found in the H1N1pdm09 viruses. Eight H7N9 virus peptides were novel, or at least not found in any of the circulating viruses in the past century, Figure 5.

Of the 10 H5N1 virus M1 peptides, two peptides (M1_17–31/27–35_) were noted in <30% of H1N1 viruses for a brief period in the 1940s, however they were novel in the H2N2, H3N2, post-1977 H1N1 and H1N1pdm09 viruses (pattern A), Figure 5. Four peptides (M1_29–57/35–49/34–55/40–57_) were found in human H1N1, H2N2, H3N2, post-1977 H1N1 and H1N1pdm09 viruses of the past century, Figure 5 (patterns B and C). One peptide (M1_29–37_) was also found in early H1N1, H2N2, H3N2 and post-1977 H1N1 viruses but was not noted in H1N1pdm09 viruses, Figure 5 (pattern C). A further two peptides (M1_95–112/99–109_) were found in <10% of viruses only in the first year of emergence of H2N2 and H3N2 viruses, they did however re-emerge in high numbers of H1N1pdm09 viruses in 2009, Figure 5 (pattern D). One peptide (M1_239–248_) was noted (>95%) in early H1N1, H2N2, post-1977 H1N1 and H1N1pdm09 viruses, however this peptide was found only in the circulating H3N2 viruses pre-1977, Figure 5 (pattern E). Peptide M1_241–252_ was present in close to 100% of viruses of all four subtypes over the last century, however in the early H1N1 viruses the number of viruses found with this peptide present varied between 80% and 100% of viruses, Figure 5 (pattern F).

All M1 peptide temporal circulation patterns are shown in Supplementary Material, Figure S4. Briefly for peptides that had the same sequences for both the H5N1 and H7N9 viruses, 7% were novel and not found in circulating human influenza viruses, 63% were found in over 90% of all human influenza viruses with the remaining peptides found in varying levels and time points in the human influenza viruses.

## Discussion

This study examined the temporal patterns of circulation of H5N1 and H7N9 viral immunogenic peptides that were present in the NP, PB1 and M1 proteins found in human influenza viruses. The peptides found in the NP protein have been studied extensively with more known regarding the characteristics of the NP peptides than those found in the PB1 and M1 proteins. The choice of the study peptides located in the NP, PB1 and M1 proteins allowed for the evaluation of our hypothesis that differences in the observed epidemiology of H5N1 and H7N9 virus cases may relate to prior exposure to homologous immunogenic peptides previously circulating in human influenza strains. Of interest were H5N1 and H7N9 virus immunogenic peptides that differed in both their sequences and temporal patterns of circulation in influenza viruses circulating in humans over the past century.

No clear relationship between exposure to past influenza viruses and susceptibility to H7N9 was observed as overall H7N9 virus peptide sequences were more novel, with the exception of two peptides (NP_169–186/170–186_) that emerged in H3N2 viruses in 1977, Figure 3. Individuals born between 1977 and 2013 whose primary exposure was to the H3N2 virus, Figure 1, may have been afforded a level of cross-protection against the H7N9 avian virus by these two peptides. The HLA type for most of those who contracted the H7N9 virus is unknown [32]; therefore it is difficult to determine the protective role of CD8+ T cells against these epitopes.

In contrast, almost all the H5N1 virus peptides of interest, in particular those which differed in both sequence and temporal circulation to the H7N9 peptides, circulated in influenza viruses isolated from humans over the past century providing opportunity for exposed individuals to retain some degree of cross-protection against the H5N1 avian virus. As the H5N1 virus peptides of interest circulated mostly between 1918 and 1990 in H1N1, H2N2 and H3N2 viruses, older birth cohorts were more likely to have this cross-protection, Figure 1. This hypothesis is consistent with the observation that H5N1 virus cases were younger (median age 26 years) than H7N9 virus cases (median age 63 years), with the infections occurring in individuals from the 1977–2009 birth cohort, with the majority (70%) born after the late 1980s when relevant peptides were no longer present in circulating H3N2 viruses. A similar age distribution was noted by Cowling *et al*. [33].

As the H7N9 virus peptides of interest had greater novelty than the H5N1 virus peptides across the three proteins, a lower level of cross-protection would be expected by circulating seasonal human influenza viruses. While this may explain the higher H7N9 case load, information about the underlying zoonotic outbreaks and subsequent human exposures is needed to confirm this hypothesis (Table 1). It was also noted that those infected with the H7N9 virus were more likely to have an underlying chronic risk condition, which may indicate that the risk of progression to disease has a greater influence on observed cases than exposure risk [34]. Furthermore, immunosenescence may have also played a role in the dominance of an older population in laboratory-confirmed H7N9 virus cases. Sentinel surveillance in comparison with hospital-based surveillance in China has suggested that the latter may have provided an incomplete picture of the extent of mild infections in the population, with infections in healthy adults more likely to be undetected [34, 35]. Mild infections of H7N9 virus were also found to be more common than H5N1 virus infections with estimates of undetected mild H7N9 virus infections in the population in the tens of thousands [36]. Severity of illness has been associated with an increase in age in patients infected with H3N2 and H1N1pdm09 seasonal viruses and this may also be the case for those infected with the H7N9 virus [37]. However, relevant data were not available to assess an age-specific risk for those with H7N9 virus infections in the sentinel surveillance system [34].

It is likely that multiple immune mechanisms have influenced the susceptibility of cohorts to influenza. For instance, HA stalk antibodies have also been linked as having an impact on the age differences noted between the cohorts who became infected with either H5N1 (group 1) or H7N9 (group 2) viruses. Primary childhood exposure to either group 1 HA or group 2 HA influenza subtype has been thought to provide lifelong protection against other hetero-subtypic group HA viruses by generation of stalk antibodies [25, 38]. However, during the 2009 H1N1 virus pandemic, caused by a group 1 HA virus, individuals with primary childhood exposure to the H2N2 virus pandemic influenza strain (also group 1 HA) experienced higher incidence of death and disease than those whose first exposure was to the group 2 HA H3N2 virus [39].

Previous studies have examined the immunogenic peptides of the H5N1 and/or H7N9 viruses for evidence of conserved peptides found in both subtypes and their relationship to those identified in previously circulating strains [21, 40]. This study extends previous studies by taking a more comprehensive approach by studying all viral sequences available for the NP, PB1 and M1 proteins from 1918 until 2013, in particular the immunogenic peptides found in these three proteins, for evidence of prior circulation in the human population. However, there are some limitations to this study, such as the low numbers of influenza viral sequences available from the early 20th century due to the lack of samples from that time-period, which may have underestimated population level diversity. Vast numbers of human HLA-1 restricted immunogenic peptides across diverse ethnic groups remain to be described. Even for many CD8+ T cell peptides, the precise HLA-restriction has not been determined. Similarly, although it is well established that mutations in antigenic peptides can result in immune evasion, functional studies by Rimmelzwaan *et al*. have demonstrated that a single mutation in the peptides NP_380–388/383–391_ results in a functional loss for the HLA types restricted by these peptides [21, 41]. The amino acid substitutions noted between the H5N1 and H7N9 peptides of interest have the potential to cause conformational changes that could impact on CD8+ T cell receptor binding if located in or close to an anchor site [41]. However, as functional studies of influenza peptide variants are few in number, the importance of the variation we observed between many H5N1 and H7N9 viral immunogenic peptides remains unknown.

Studies have shown evidence of T cell cross-protection between human influenza subtypes [19] including seasonal and pandemic influenza strains [20, 42]. Our data describing differences in the temporal circulation patterns of the immunogenic peptides identified in H5N1 and H7N9 viruses accord with the observation that a lower proportion of H5N1 virus cases has been observed in older individuals, possibly due to prior exposure to related peptides. The reason for the greater number of H7N9 virus cases among the elderly is less clear from these data, but may have some relationship with the circulation of two related NP peptides in H3N2 viruses in the latter part of the 20th century. Development of broadly cross-protective influenza vaccines capable of eliciting immunity against emerging viruses remains a challenging goal. Studies such as ours may help provide insights into mechanisms contributing to cohort cross-protection and aid identification of suitable candidate antigens.

## Supplementary material

For supplementary material accompanying this paper visit https://doi.org/10.1017/S095026881900102X. click here to view supplementary material
