# Supplementary figures and images for: Prior exposure to immunogenic peptides found in human influenza A viruses may influence the age distribution of cases with avian influenza H5N1 and H7N9 virus infections
Source: Epidemiol Infect. 2019 Jun 13;147:e213. doi: 10.1017/S095026881900102X (PMC6624876; doi:10.1017/S095026881900102X)

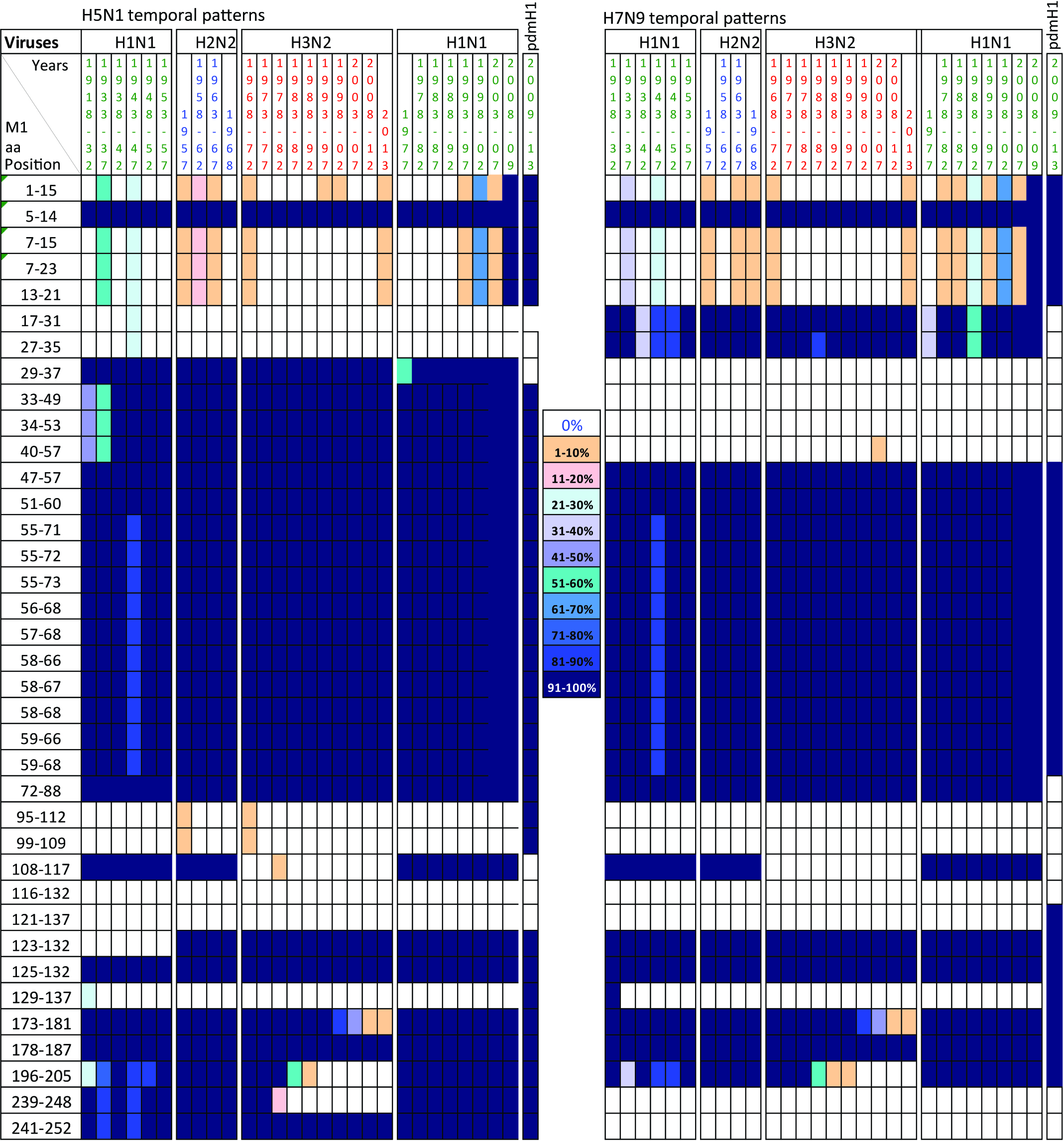

Supplement: Supplementary file 1 [file S095026881900102Xsup001.zip › S095026881900102Xsup001/Supp-Figure_S4.jpg]

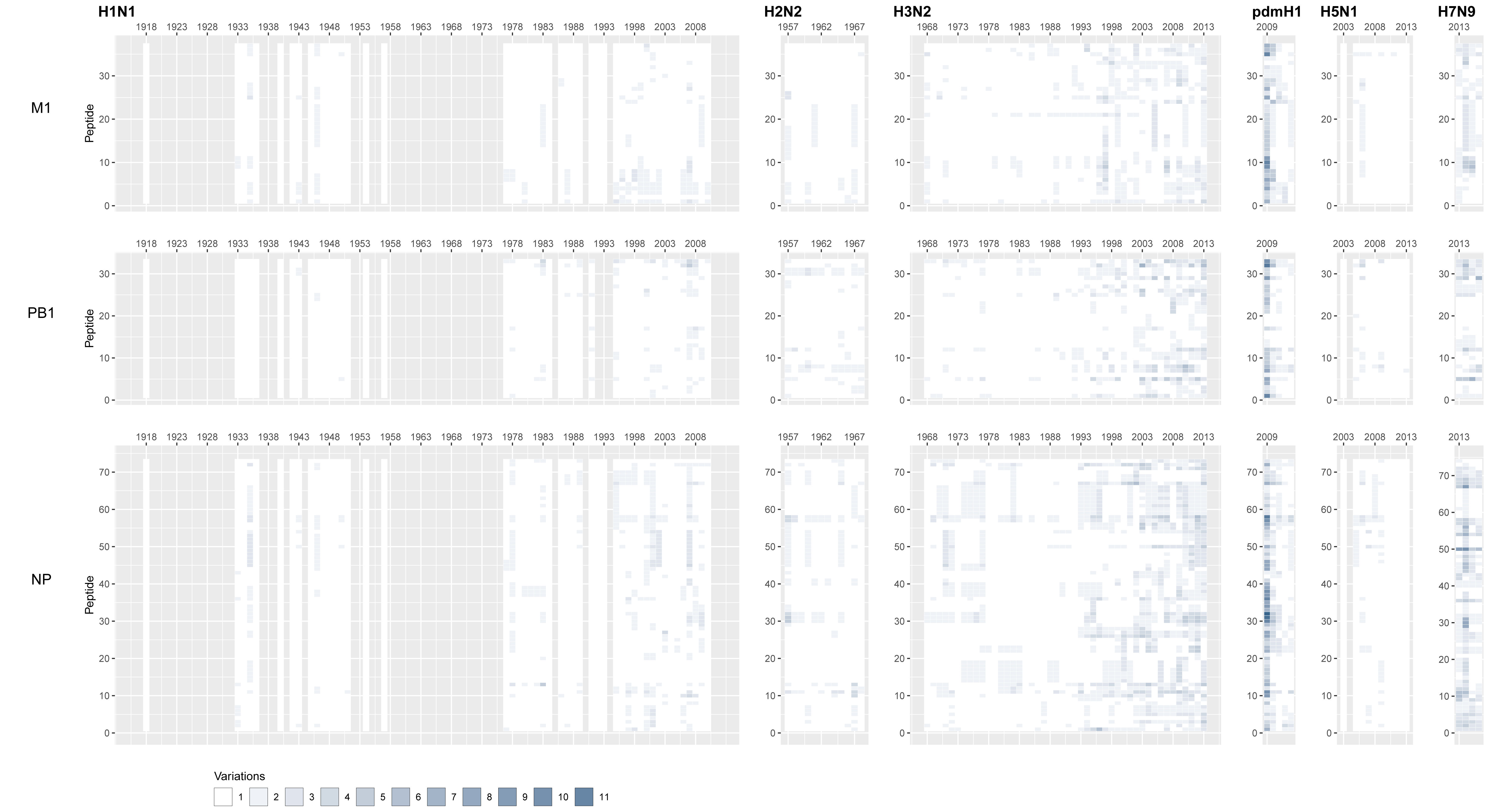

Supplement: Supplementary file 1 [file S095026881900102Xsup001.zip › S095026881900102Xsup001/Supp_Figure S1.jpg]

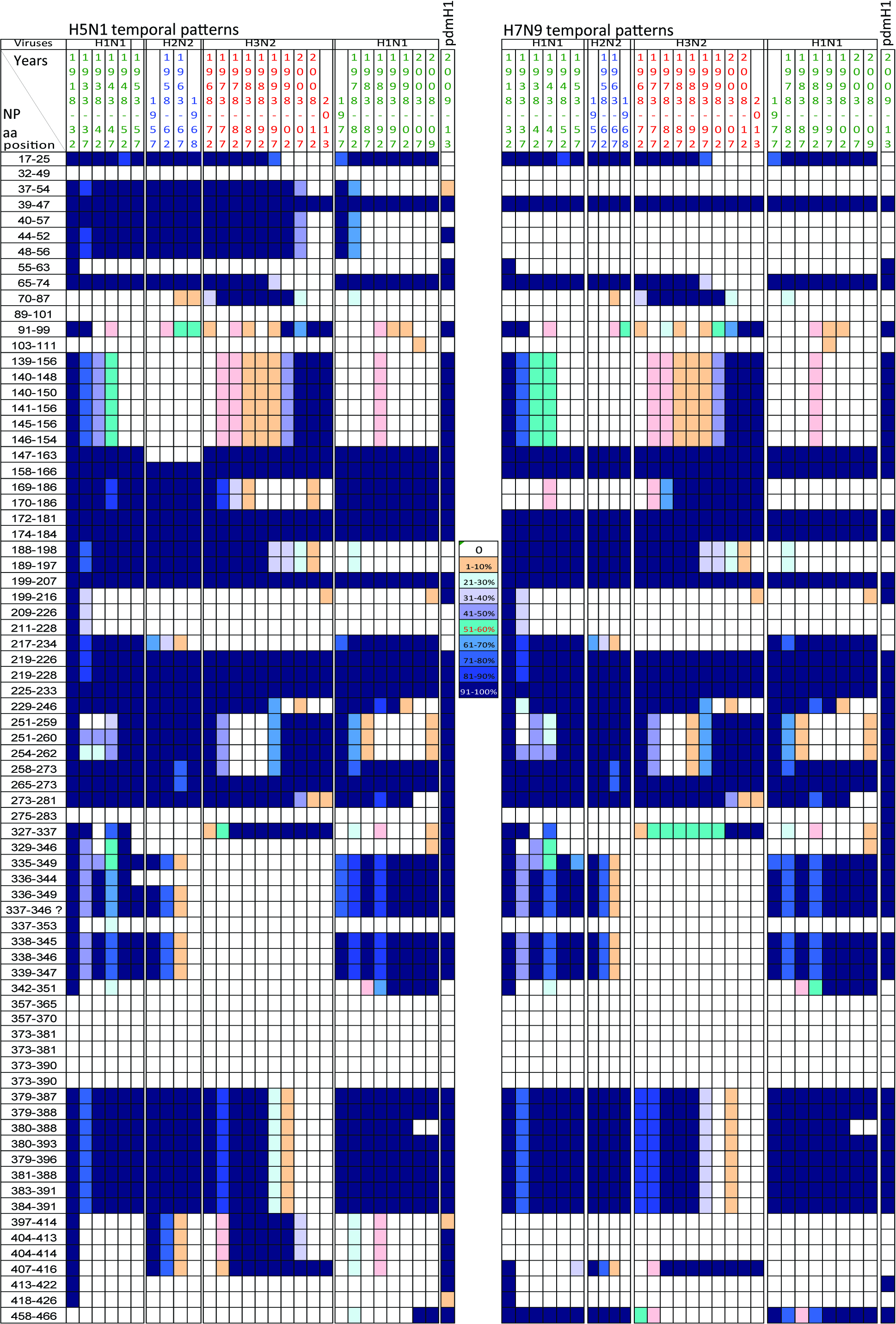

Supplement: Supplementary file 1 [file S095026881900102Xsup001.zip › S095026881900102Xsup001/Supp_Figure_S2.jpg]

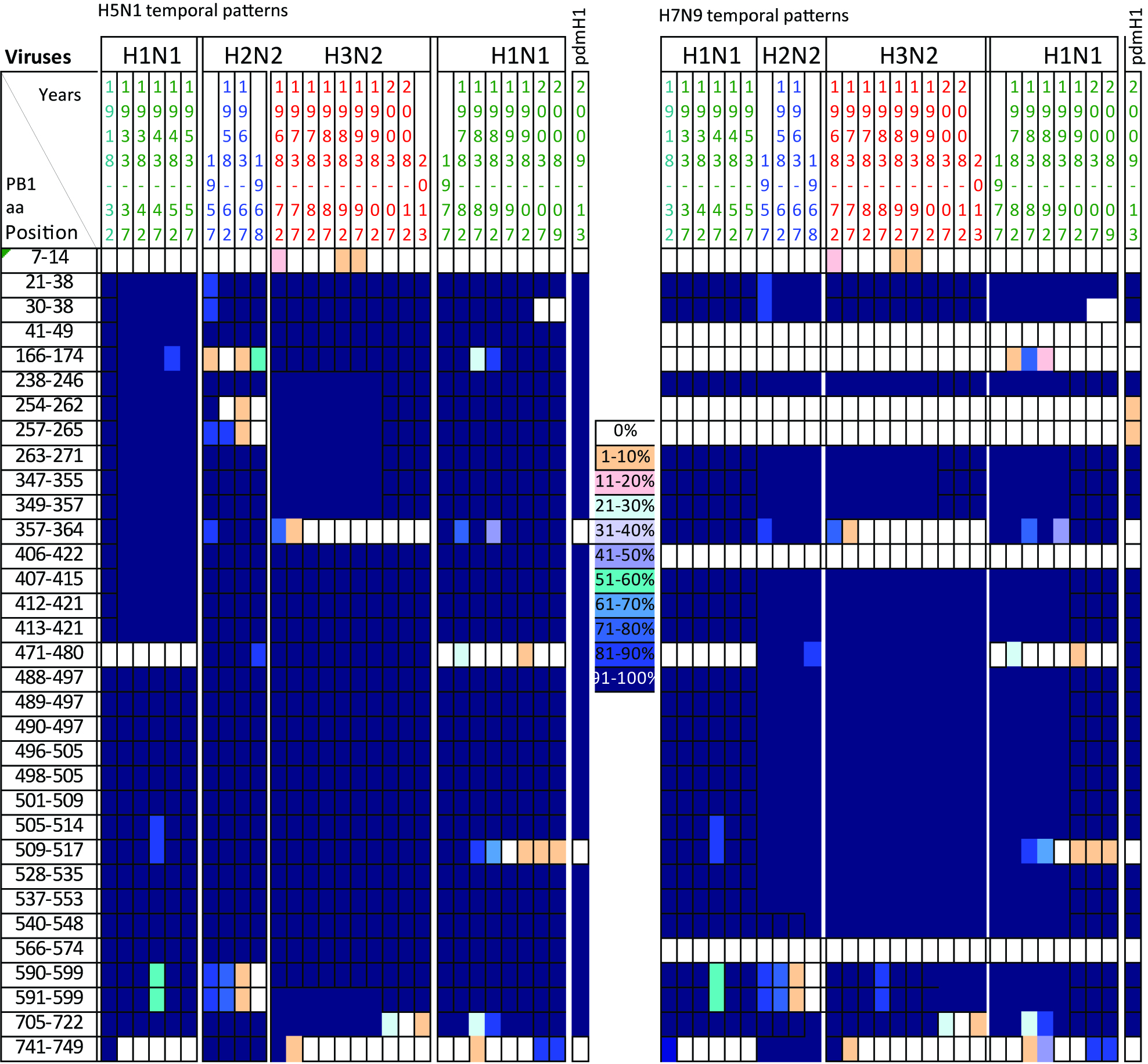

Supplement: Supplementary file 1 [file S095026881900102Xsup001.zip › S095026881900102Xsup001/Supp_Figure_S3.jpg]
